# Implant Failure Rate in Transcrestal and Lateral Window Sinus Augmentations: A Descriptive Retrospective Cohort Study

**DOI:** 10.3390/dj14070409

**Published:** 2026-07-06

**Authors:** Eran Gabay, Yuval Shafran, Tarek Mtanis, Eli E. Machtei, Ofir Ginesin, Hadar Zigdon-Giladi, Yaniv Mayer

**Affiliations:** 1 Department of Periodontology and Implant Dentistry, Rambam Health Care Campus, Haaliya Hashniya 8, Haifa 3109601, Israel; dr.tarekmtanis@gmail.com (T.M.); o_ginesin@rambam.health.gov.il (O.G.); h_zigdon@rambam.health.gov.il (H.Z.-G.); yaniv.mayer@technion.ac.il (Y.M.); 2 Rappaport Faculty of Medicine, Technion Institute of Technology, Haifa 3109601, Israel; yuvalshafran@campus.technion.ac.il

**Keywords:** sinus augmentation, dental implants, transcrestal approach, lateral window approach

## Abstract

**Objectives:** This study aimed to describe implant survival rates and associated risk factors in patients treated with two sinus augmentation techniques—Lateral Window (LW) and Transcrestal (TC)—at a single center. **Methods:** A retrospective cohort analysis was performed using medical records of patients who underwent maxillary sinus augmentation and subsequent dental implant placement between 2015 and 2019 at the Rambam Healthcare Center. Data on demographic variables, surgical approach, graft materials, implant characteristics, and clinical outcomes were collected from electronic medical records. **Results:** A total of 135 patients met the inclusion criteria, contributing 144 TC procedures (with 144 implants) and 46 LW procedures (90 implants) to the final analysis. The overall implant failure rate was 7.7%, with a significantly higher failure rate for TC (11.11%) compared to LW (2.22%). Procedure type was a significant predictor of implant survival (OR 1.62); however, as residual bone height (RBH) data were unavailable, confounding by indication cannot be excluded and this finding should be interpreted with caution. Implant length demonstrated a significant influence on outcomes (OR 1.28 per mm increase); shorter implants (8 mm) exhibited lower survival rates (81.3%) compared to 10 mm implants (92.3%). Smoking status was identified as a negative predictor of implant survival (OR 0.23). Schneiderian membrane ruptures occurred in nine cases, seven of which were LW procedures. Smoking was the only factor affecting graft survival (OR 0.29). **Conclusions:** In this retrospective cohort, implant survival rates were higher in the LW group than in the TC group; however, given that individual residual bone height data were unavailable and case assignment, although governed by standardized departmental criteria, was not randomized, the possibility of confounding by indication cannot be excluded. Smoking and shorter implants were significant risk factors for implant failure. Smoking was also associated with graft failure.

## Simple Summary

What is known: Maxillary sinus floor elevation (MSFE) is a well-established procedure with high success rates documented in the dental literature for implant placement in the posterior maxilla with insufficient bone height. Two main approaches exist—Lateral Window (LW) and Transcrestal (TC)—each with distinct advantages and limitations. What this study adds: This large-scale retrospective cohort study reports higher implant survival rates in the LW group (97.8%) compared to the TC group (88.9%). However, because residual bone height data were not available, and residual bone height is a primary determinant of technique selection, this difference should be interpreted as an observed association rather than evidence of technique superiority. Smoking status and shorter implant length were identified as significant risk factors for implant failure.

## 1. Introduction

Over the past three decades, implant-supported prostheses have provided a predictable treatment option with a high success rate [1]. However, due to alveolar ridge resorption and maxillary sinus pneumatization following tooth extraction, lack of sufficient bone height for implant placement is a common clinical challenge in the posterior maxilla [2,3,4]. Therefore, bone augmentation procedures such as maxillary sinus floor elevation (MSFE) are advised for optimal implant placement and high mid- to long-term implant survival rate [5,6,7,8]. Alternatively, short, tilted, and zygomatic implants have been introduced as adjunctive options [9,10].

MSFE is a predictable treatment approach with a high success rate [11]. It involves entering the maxillary sinus cavity, displacing the Schneiderian membrane and apically elevating the sinus floor with bone substitute material, which can be either autogenous or xenogenous. Based on the RBH, clinical experience and judgment, two main MSFE techniques are available, which are well documented and provide acceptable clinical outcomes. The Transcrestal (TC) approach, which requires simultaneous implant placement, is considered less invasive and has shorter healing time [12,13]. The sinus floor is elevated through a small osteotomy created in the alveolar ridge using various instruments. However, the absence of direct surgical visualization represents a notable limitation of this technique. The Lateral Window (LW) approach does not require simultaneous placement but is considered invasive with higher complication and morbidity rates [14,15]. Sinus membrane elevation and bone augmentation are performed through a lateral window prepared on the buccal aspect of the alveolar process. This direct approach allows inspection of membrane integrity and more precise bone augmentation.

MSFE usually requires bone grafting materials for endo-sinus new bone formation. The use of autogenous bone (AB) is still considered the gold standard in bone grafting due to its superior biological advantages such as osteogenic, osteoconductive, and osteo-inductive properties. However, its high resorption rate and the need for a donor site limit its use. Deproteinized bovine bone mineral (DBBM) is commonly used, as it possesses osteoconductive properties and provides a scaffold for new bone formation. A 5-year implant survival rate of 95% was reported following MSFE performed with DBBM [16].

Dental implant failures may be classified into early and late implant failures [17]. Early implant failures (EIFs) occur prior to prosthetic loading, whereas late failures are defined as those occurring after functional loading has been initiated. However, there is no specific consensus on the precise definition for the timing of EIF. While studies have shown EIF to primarily occur 1–2 years after implantation, EIF in augmented maxillary sinus bone may be classified based on timing—either within the first 6 months after implant placement or up to 12 months following functional loading [18]. Despite the abundant literature on MSFE, implant failure and survival rates, and risk factors affecting the survival and success of dental implants, studies specifically investigating the effect of MSFE approach on implant failure rate before and after functional loading are lacking [19]. Furthermore, factors such as implant placement timing (simultaneous vs. delayed), type of graft material, and patient-related variables (e.g., smoking, systemic conditions) may also impact outcomes [20,21].

This retrospective study was undertaken to further evaluate and compare implant failure rate following two different methods of sinus augmentation. This study describes implant failure rates following LW and TC sinus augmentation procedures and explores patient- and implant-level factors associated with survival outcomes. Additionally, it is expected that the TC approach will lead to fewer intraoperative and postoperative complications.

## 2. Materials and Methods

### 2.1. Data Collection and Study Design

Data collection for this retrospective study was conducted between 27 April 2023 and 21 January 2024. To ensure a minimum follow-up period of 5 years, eligible patient records were identified using institutional registry codes for LW and TC sinus augmentation procedures performed between 1 January 2015 and 31 December 2019 at the Department of Periodontology, Rambam Healthcare Center. The study complied with the principles of the Declaration of Helsinki and was approved by the Ethics Committee of Rambam Healthcare Center (Haifa, Israel) (Reference: 0099-23-RMB-D; approval date: 27 April 2023).

Patient medical records were reviewed until one of the following endpoints was reached: successful implant integration followed by referral for prosthetic rehabilitation without subsequent follow-up, implant failure after prosthetic restoration, EIF, or loss to follow-up without documented evidence of implant failure. The study cohort comprised patients who underwent single or multiple maxillary sinus augmentation procedures.

The inclusion criteria were as follows: (1) patients aged > 18 years, (2) patients who underwent sinus augmentation and dental implants placement in the same site, (3) documented follow-up for at least 6 months or EIF occurring before 6 months, (4) adequate primary stability. Exclusion criteria included: (1) use of Poly L-lactide-co-ε-caprolactone-coated bovine bone graft, a bone substitute that underwent a recall process during that period [21]; (2) patients who underwent a second sinus augmentation in the same position due to prior failure; (3) insufficient data regarding any demographic or clinical parameters collected in the study.

### 2.2. Data Retrieval and Management

Patient data were retrieved from “Prometheus,” an electronic medical record (EMR) system developed at Rambam Hospital. Supplementary data were obtained from manually documented clinical records. The dataset included demographic details (age, gender, and smoking status, reported as yes/no in the anamnesis) and clinical information pertinent to the sinus augmentation procedure. This clinical data encompassed the type of surgery performed (LW/TC), the volume and size of graft particles used, the type of administered antibiotics, and intra- and postoperative complications. Furthermore, data on dental implant characteristics, such as dimensions and position, and records of dental implant failures were systematically recorded. Cases with missing data that could not be retrieved through thorough manual documentation searches were entirely excluded from the dataset.

### 2.3. Treatment Procedures

All surgical procedures were conducted at the Department of Periodontology, Rambam Healthcare Center. All surgical procedures were performed either by postgraduate residents under the direct supervision of an experienced periodontist, or by an attending periodontist. The subsequent implant restoration process was completed at the prosthetic department, a private clinic, or a public dental clinic.

Surgical approach selection was based on the treating clinician’s judgment at the time of surgery, informed by assessment of sinus anatomy on preoperative CBCT, predictable RBH, and patient-specific factors. In the absence of a standardized allocation protocol, TC was generally favored when RBH was considered sufficient for simultaneous implant placement, while LW was typically selected for cases requiring greater augmentation volume or more complex anatomy. These clinical decisions were decided according to the department of periodontology guidelines specifying selection criteria for each surgical approach (summarized in Table 1).

However, because RBH measurements were not systematically recorded in the institutional database, formal analysis of allocation criteria was not possible in this study.

### 2.4. Outcome Measurement

The primary outcome of this study was the failure rate of implants placed during or following MSFE. Implant failure was defined as loss of osseointegration or implant removal for any reason, including persistent suppuration, or extensive radiographic bone loss.

Secondary outcome measures included EIF, dental implant survival rate, graft survival rate, and complication rates, such as Schneiderian membrane perforations, sinusitis, and secondary infections. EIF was defined as implant loss within the first 18 months after implant placement. Specifically, implant loss within the first 6 months after placement was attributed to osseointegration failure, whereas early implant loss during the first 12 months after loading was considered a consequence of overloading. Implant survival was documented for any implant that remained in the oral cavity with its supporting restoration at the most recent recall appointment and showed no indication for removal. The current study focused on documented implant failures within the cohort along the overall follow-up period. However, there was a possibility that some failures, especially in the long-term follow-up period, were treated in the community and were not documented in the hospital EMR.

### 2.5. Data Management and Statistical Analyses

Statistical analysis was performed using R statistical software (version 4.2.3; R Foundation for Statistical Computing, Vienna, Austria). Specialized packages employed included ‘lme4’ for mixed-effects logistic regression models and ‘survival’ for time-to-event analyses.

Demographic and procedural data were analyzed using descriptive statistics, including means with standard deviations for continuous variables and frequencies with percentages for categorical variables. The distribution of implant dimensions across the study population was analyzed, specifically implant diameters (3.3 mm, 3.5 mm, 3.75 mm, 4.2 mm, and 5 mm) and lengths (8 mm, 10 mm, and 11.5 mm).

The primary outcome measures, implant and graft survival rates, were calculated for each procedural group and stratified by relevant patient factors. Differences between groups were assessed using Student’s *t*-test for continuous variables and chi-square or Fisher’s exact test for categorical variables, as appropriate. To account for the clustering effect of multiple implants within the same patient or sinus, mixed-effects logistic regression models were employed with patient ID as a random effect.

Time-dependent failure analysis was conducted using Kaplan–Meier survival curves and log-rank tests to compare survival distributions. Failures were categorized as healing period failures (0–6 months), early loading failures (6–18 months), and late failures (>18 months).

Multivariate logistic regression analysis was performed with implant survival and graft survival as dependent variables to identify key factors influencing survival outcomes. Independent variables included procedure type (LW vs. TC), patient age, gender, smoking status, bone graft type, and implant dimensions (diameter and length). The regression models generated odds ratios (ORs) with 95% confidence intervals (CIs) to quantify the strength of association between predictors and outcomes.

Model fit was assessed using the Hosmer–Lemeshow goodness-of-fit test and the Akaike Information Criterion (AIC). A *p*-value of <0.05 was considered statistically significant.

### 2.6. Sample Size and Power Calculation

A priori power calculation was performed to detect a clinically meaningful difference in implant survival between the TC approach (reported 3-year survival rate: 92.8%). and the LW approach (reported 3-year survival rate: 98.3%), according to a systematic review by Pjetursson et al. (2008) [11]. The calculation set an alpha error of 5% and a beta error of 0.20 (power of 80%) to determine the minimal cohort size of 101 implants.

This study was conducted in compliance with STROBE guidelines recommendations for observational studies.

## 3. Results

### 3.1. Patient Demographics and Exclusions

**Figure 1:**
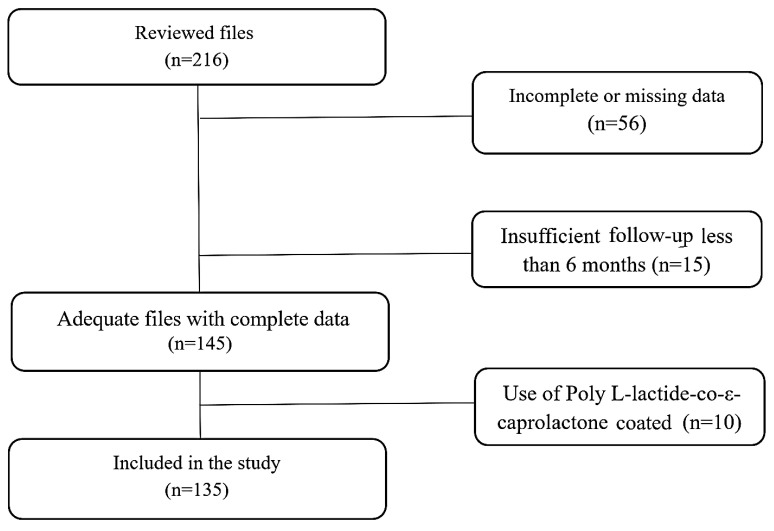
Flow chart of the study’s data set acquisition. A total of 216 patient files were reviewed for eligibility. Of these, 56 files were excluded due to incomplete or missing data regarding the procedure and another 15 were excluded due to insufficient documented follow-up of less than 6 months. An additional 10 cases were excluded because of the use of Poly L-lactide-co-ε-caprolactone-coated bovine bone grafts. The final cohort included 135 cases, who met the inclusion criteria.

A total of 216 patient electronic medical records (EMRs) were identified and reviewed for eligibility. Of these, 71 records were excluded for various reasons: 56 due to incomplete or missing procedural data, 10 because of the use of Poly L-lactide-co-ε-caprolactone-coated bovine bone grafts, and 15 due to insufficient documented follow-up of less than six months. Ultimately, 135 patients met the inclusion criteria and were included in the final analysis (Figure 1). Patients were followed for up to 75 months; follow-up was terminated at the time of implant loss.

### 3.2. Surgical Procedures and Outcomes

A total of 190 sinus augmentation procedures were performed. Of these, 144 were TC procedures, and 46 were LW procedures. Among the LW procedures, 32 (69.57%) were performed as one-stage procedures, and 14 (30.43%) as two-stage procedures.

Membrane ruptures occurred in nine cases (4.74% of total procedures), with seven of these noted following LW procedures (15.22% of LW cases). Antibiotics were prescribed in all cases: Amoxicillin or Augmentin was used in 78.2% of procedures, while Clindamycin or Erythromycin was used in 21.8% of cases for penicillin-sensitive patients.

A total of 234 implants were placed: 144 implants (61.53%) following TC sinus augmentation procedures and 90 implants (38.46%) following LW sinus augmentation procedures (Table 2). Implant diameter and length distributions were comparable between the two sinus augmentation groups.

A total of 18 implant failures (7.7%) were recorded among 16 patients, with two patients experiencing additional failures on the contralateral side. Of the 18 implant failures, 16 occurred in the TC group, corresponding to a failure rate of 11.11% in that group. In contrast, only two failures occurred in the LW group, representing a failure rate of 2.22%. This difference between the two procedures was statistically significant (*p* = 0.011).

**Figure 2:**
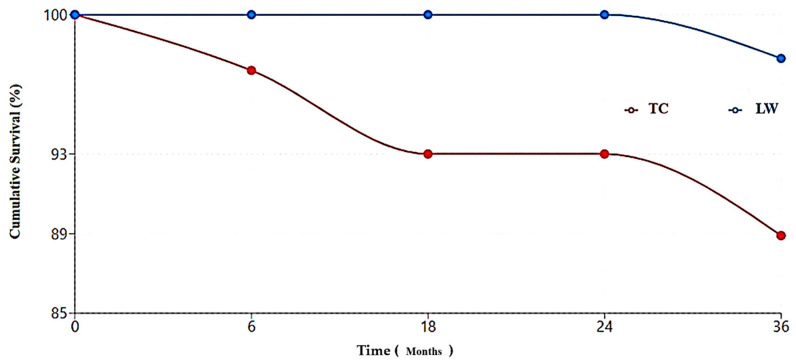
Implant survival over time. Kaplan–Meier survival curves demonstrate time-dependent implant failures. LW procedures maintained 100% implants survival rate for the first 24 months, while TC procedures showed implant failures in each time period: healing period failures (0–6 months), early loading failures (6–18 months), and late failures (>18 months).

Pre-loading failures (within the 6-month healing period) occurred in four cases (1.71%). Early failures (within one-year post-loading) occurred in an additional six cases (2.56%). Another eight implants (3.42%) were lost after more than 24 months post-surgery (Table 3). Notably, two of the eight implants lost during the late failure period (>24 months) had been installed via the LW procedure (Figure 2).

### 3.3. Regression Analysis and Risk Factors

Logistic regression analysis showed an association between procedure type and implant survival (OR 1.62, 95% CI 1.21–23.9, *p* = 0.027), with implant survival being higher in the LW group than in the TC group (Table 4).

Implant length also significantly influenced outcomes (OR 1.28 per mm increase, 95% CI 1.03–1.59, *p* = 0.038). Shorter implants (8 mm) showed significantly lower survival rates (81.3%) compared to 10 mm implants (92.3%, *p* = 0.023).

Smoking status was identified as a negative predictor of implant survival (OR 0.23, 95% CI 0.08–0.66, *p* = 0.006). When examining the combined effect of procedure type and smoking status on implant survival outcomes, non-smokers exhibited higher survival rates: 87.02% (114/131) in the TC procedures and 93.83% (76/81) in the LW procedures. In contrast, smokers demonstrated much lower survival rates: 69.23% (9/13) for TC and 66.67% (6/9) for LW (Table 5).

Logistic regression analysis found that smoking was the only factor significantly affecting graft survival (OR 0.29, 95% CI 0.09–0.95, *p* = 0.041). The other two predictors for implant survival, procedure type (TC/LW) and implant length, had no significant effect on graft survival (Table 6).

## 4. Discussion

In the present cohort, implant survival rates were higher in the LW group (97.78%) than in the TC group (88.89%). Of the 18 total implant failures, 16 were associated with TC procedures. The lower failure rate observed in the LW group is consistent with previous studies reporting high predictability for this approach in cases with greater bone loss [11,22]. Our findings align with findings by Del Fabbro et al. (2008), who reported survival rates of 92% to 98% in a systematic review of LW sinus augmentations [23]. More recently, a 15-year retrospective study by Jamcoski et al. (2023) [24] reported an implant success rate of 97.2% when the RBH was ≥4 mm. In contrast, the TC approach has shown more variability in implant survival. Tan et al. (2008) found survival rates ranging from 85% to 95% depending on the degree of RBH [12], while Ragucci et al. (2019) demonstrated that implants penetrating the sinus cavity using minimally invasive techniques achieved a weighted mean survival rate of 95.6%, particularly when the RBH exceeded 4 mm [25]. The 88.89% survival rate observed in this study for the TC approach reflects its limitations in severely resorbed maxillae. According to the literature, the LW approach offers greater visibility, control, and bone volume, which may contribute to its reported predictability [26,27]. The LW approach provides direct visualization of the Schneiderian membrane, allowing for careful elevation and precise graft placement. This advantage may be particularly important in cases with complex sinus anatomy or limited RBH. Starch-Jensen et al. (2017) highlighted that LW sinus augmentation provides dependable long-term outcomes, particularly in cases with substantial bone loss [16]. Conversely, the TC approach, while less invasive, suffers from limited visibility. As noted by Summers (1994), while membrane perforations are less common with this technique, inadequate elevation can lead to insufficient bone volume for stable implant placement, thereby increasing the risk of late implant failure [28].

Most implant failures occurred following prosthetic loading rather than during the initial healing period. Overall, pre-loading failures (within the 6-month healing period) occurred in only four cases (1.71%), while early failures (within one year post-loading) occurred in six cases (2.56%). An additional eight implants (3.42%) were lost after more than 24 months post-surgery. This pattern suggests that the underlying cause of failure may be related to compromised osseointegration rather than immediate surgical complications [29]. A potential explanation for the higher failure rate after loading is the lower percentage of vital bone in the augmented sinus, resulting from non-resorbing xenogenic bone graft material that occupies a significant portion of the augmented volume. The reduced proportion of vital bone in the peri-implant region may impair the bone-to-implant contact (BIC), thereby compromising the long-term success of the implant. Supporting this notion, histologic analysis of biopsies taken from rabbit sinuses augmented with xenogeneic bone graft revealed 34% of vital bone after 8 weeks and a relatively low BIC of 26% [30].

Smoking status significantly affected implant survival in both procedural groups. Smokers exhibited markedly lower survival rates (69.23% and 66.67% respectively), compared to non-smokers (87.02% and 93.83% respectively). Logistic regression analysis confirmed that smoking was a significant risk factor for implant failure. These findings align with prior studies describing compromised healing and osseointegration in smokers [31,32,33]. Smoking is known to impair wound healing by reducing blood flow to the surgical site [34], decreasing oxygenation of healing tissues [35], and inhibiting immune response [36]. The combined detrimental effect of smoking was pronounced in the TC and LW groups. By compromising healing, smoking may have a negative effect on the osseointegration process. A recent multicenter nested case–control study strengthened this assumption by demonstrating that current smoking was found as a prominent risk factor for early implant failure, both in LW and TC procedures [37]. Furthermore, smoking may exert a long-term detrimental effect on implant survival in MSFE sites. A recent meta-analysis, which was focusing on the predictive factors of LW procedure long-term outcomes, reported that smoking significantly impaired implants survival in a 5–13 years follow-up [38].

Implant length was positively associated with survival, suggesting that longer implants offer improved primary stability and surface area for osseointegration. Shorter implants (8 mm) showed significantly lower survival rates (81.3%) compared to 10 mm implants (92.3%). These findings are consistent with biomechanical principles suggesting that increased implant length provides greater surface area for osseointegration and improved resistance to occlusal forces [39,40]. Implant diameter, however, did not affect outcomes. This finding is consistent with prior findings indicating limited influence on long-term implant survival [41,42]. This observation suggests that implant length may be a more critical factor than diameter when planning implant placement in augmented sinuses.

Complications such as Schneiderian membrane rupture, graft infection, and sinusitis remain key concerns in sinus augmentation procedures. In this study, Schneiderian membrane rupture occurred in nine cases (4.74% of total cases), with seven of these occurring in the LW group (15.22%). This higher rate in the LW group is consistent with the meta-analysis by Schiavo-Di Flaviano et al. (2024), which reported membrane rupture rates ranging from 7% to 56% in LW procedures [43]. However, studies by Testori et al. (2020) emphasize that these complications can often be managed intraoperatively without significant impact on implant survival, provided the rupture is minor and does not lead to sinus infection [44]. The fact that the LW group in our study maintained a high implant survival rate despite more frequent membrane perforations supports this observation, Schneiderian membrane perforations did not compromise implants survival.

Other patient-related factors, including age, gender, and comorbidities (diabetes, hypertension), did not show significant associations with implant survival. These results align with the literature reporting that well-controlled systemic conditions may not strongly impact implant success in carefully managed clinical settings [1,45].

The retrospective design of this study, together with a number of potential confounding factors, including operator experience and variability in graft materials, limits the generalizability of the findings. A main drawback was the insufficient and inconsistent documentation in the reviewed medical files, which resulted in the exclusion of 71 cases from the original data set of 216 reviewed files. An additional limitation relates to the retrospective nature of RBH assessment. Although departmental guidelines specify RBH thresholds for technique selection (Table 1), RBH values were determined by the treating clinician at the time of surgery based on CBCT review and were not entered as a discrete, standardized data field in the medical record. Retrospective re-measurement of RBH from archived imaging was not feasible for the full cohort, both due to incomplete imaging availability for excluded/early cases and the risk of measurement bias if performed non-blinded. Consequently, while RBH-based allocation criteria existed, their actual application to each individual case in this cohort could not be verified, and residual confounding by indication remains a key limitation of this study. As a result, the observed difference in implant survival between the two groups cannot be attributed to technique efficacy alone. The comparison between techniques should therefore be interpreted only as an observed association within this cohort, not as evidence of superiority of one approach over the other. Future studies should prospectively record RBH and incorporate it into both the allocation protocol and the multivariable model.

In conclusion, in this retrospective descriptive cohort, implant survival rates were higher in the LW group than in the TC group; however, given that individual residual bone height data were unavailable and case assignment, although governed by standardized departmental criteria, was not randomized, the possibility of confounding by indication cannot be excluded. This finding should therefore not be interpreted as evidence of technique superiority. Smoking and shorter implant length were independently associated with implant failure and represent clinically meaningful risk factors to consider when planning sinus augmentation procedures. These findings may inform clinical awareness of patient-specific risk factors in sinus augmentation, but prospective studies with systematic case-level documentation are needed before reliable comparative conclusions can be drawn.

## Figures and Tables

**Table 1:** Standardized guidelines specifying selection criteria for sinus augmentation approach.

Variable	TC	LW (1-Stage)	LW (2-Stage)
Sinus infection	No signs	No signs	No signs
Minimal RBH	3 mm	3 mm	≤2 mm
Maximal RBH	8 mm	NA	NA
Minimal alveolar ridge width	5 mm	5 mm	NA
Sinus floor inclination	≥45 degrees	NA	NA
Bone density	I–III	I–III	III–IV
Dental implant initial stability	≥30 N	≥30 N	NA
Maximal number of implants	2 implants	NA	NA
Size of Schneiderian membrane perforation	NA	≤5 mm	>5 mm

**Table 2:** Demographic and clinical characteristics (patient-based analysis).

Characteristic	Transcrestal	Lateral Window	Total	*p*-Value
Patients, *n*	83	52	135	
Number of sites	144	46	190	
Number of implants	144	90	234	
Age Means ± SD (range)	58.2 ± 12.3 (40–79)	59.1 ± 11.5 (40–77)	58.5 ± 12.0 (40–79)	0.671
Male/female ratio	36.1/63.9	34.6/65.4	35.6/64.4	0.854
Non-smokers Smokers	76 (91.6%) 7 (8.4%)	47 (90.4%) 5 (9.6%)	123 (91.1%) 12 (8.9%)	0.812
Diabetes	10 (12.0%)	7 (13.5%)	17 (12.6%)	0.809
Hypertension	18 (21.7%)	12 (23.1%)	30 (22.2%)	0.852

Demographic and clinical characteristics were compared between TC and LW groups using the independent samples *t*-test for continuous variables and chi-square test for categorical variables.

**Table 3:** Time-dependent implant failures by procedure type.

Procedure Type	Total Implants	Total Failures	Pre-Loading Failures (0–6 Months)	Early Post Loading Failures (6–18 Months)	Late Failures (>24 Months)
Transcrestal	144	16 (11.1%)	4 (2.8%)	6 (4.2%)	6 (4.2%)
Lateral Window	90	2 (2.2%)	0 (0.0%)	0 (0.0%)	2 (2.2%)
Total	234	18 (7.7%)	4 (1.7%)	6 (2.6%)	8 (3.4%)
*p*-Value		0.011 *	0.112	0.052	0.411

Fisher’s exact test was used to compare time-dependent failure rates between TC and LW procedures across different time periods. * Statistically significant (*p* < 0.05).

**Table 4:** Logistic regression analysis for factors affecting implant survival.

Variable	Odds Ratio	95% CI	*p*-Value
Procedure (Lateral Window vs. Transcrestal)	1.62	1.21–23.9	0.027 *
Age (per year)	1.01	0.98–1.05	0.421
Gender (female vs. male)	1.14	0.54–2.42	0.726
Smoking (vs. non-smoking)	0.23	0.08–0.66	0.006 *
Implant diameter (per mm increase)	1.21	0.45–3.24	0.712
Implant length (per mm increase)	1.28	1.03–1.59	0.038 *

Multivariate logistic regression analysis was performed to identify significant predictors of implant survival with the Wald test determining significance of individual factors. * Statistically significant (*p* < 0.05).

**Table 5:** Implant survival rates by procedure type and smoking status.

Procedure	n	Smoking Status	Implant Survival Rate	*p*-Value
Transcrestal	131	Non-smoker	114/131 (87.0%)	0.099
	13	Smoker	9/13 (69.2%)	
Lateral Window	81	Non-smoker	76/81 (93.8%)	0.031 *
	9	Smoker	6/9 (66.7%)	

The combined effect of procedure type and smoking status on implant survival outcomes. Fisher’s exact test was used. * Statistically significant (*p* < 0.05).

**Table 6:** Logistic regression analysis for factors affecting graft survival.

Variable	Odds Ratio	95% CI	*p*-Value
Procedure (Lateral Window vs. Transcrestal)	1.75	0.87–3.52	0.116
Age (per year)	1.00	0.97–1.03	0.937
Gender (female vs. male)	0.98	0.40–2.42	0.965
Smoking (vs. non-smoking)	0.29	0.09–0.95	0.041 *
Implant diameter (per mm increase)	1.32	0.51–3.43	0.569
Implant length (per mm increase)	1.11	0.88–1.39	0.384
Membrane rupture (vs. no rupture)	0.41	0.12–1.36	0.145

Multivariate logistic regression analysis was performed to identify significant predictors of graft survival with the Wald test determining significance of individual factors. * Statistically significant (*p* < 0.05).

## Data Availability

The datasets generated and analyzed during this study are not publicly available due to restrictions imposed by institutional privacy policies protecting patient medical records. However, the data may be available from the corresponding author upon reasonable request and with appropriate ethical approvals.

## References

[1] Buser D., Sennerby L., De Bruyn H. (2017). Modern implant dentistry based on osseointegration: 50 years of progress, current trends and open questions. Periodontology 2000.

[2] Schropp L., Wenzel A., Kostopoulos L., Karring T. (2003). Bone healing and soft tissue contour changes following single-tooth extraction: A clinical and radiographic 12-month prospective study. Int. J. Periodontics Restor. Dent..

[3] Cavalcanti M.C., Guirado T.E., Sapata V.M., Costa C., Pannuti C.M., Jung R.E., Neto J.B.C. (2018). Maxillary sinus floor pneumatization and alveolar ridge resorption after tooth loss: A cross-sectional study. Braz. Oral Res..

[4] Hameed M.H., Gul M., Ghafoor R., Khan F.R. (2019). Vertical Ridge Gain with Various Bone Augmentation Techniques: A Systematic Review and Meta-Analysis. J. Prosthodont..

[5] Jung R.E., Zembic A., Pjetursson B.E., Zwahlen M., Thoma D.S. (2012). Systematic review of the survival rate and the incidence of biological, technical, and aesthetic complications of single crowns on implants reported in longitudinal studies with a mean follow-up of 5 years. Clin. Oral Implants Res..

[6] Barone A., Orlando B., Cingano L., Marconcini S., Derchi G., Covani U. (2012). A randomized clinical trial to evaluate and compare implants placed in augmented versus non-augmented extraction sockets: 3-year results. J. Periodontol..

[7] Papaspyridakos P., De Souza A., Vazouras K., Gholami H., Pagni S., Weber H.-P. (2018). Survival rates of short dental implants (≤6 mm) compared with implants longer than 6 mm in posterior jaw areas: A meta-analysis. Clin. Oral Implants Res..

[8] Park W.-B., Kang K.L., Han J.-Y. (2019). Factors influencing long-term survival rates of implants placed simultaneously with lateral maxillary sinus floor augmentation: A 6- to 20-year retrospective study. Clin. Oral Implants Res..

[9] Esposito M., Cannizzaro G., Soardi E., Pistilli R., Piattelli M., Corvino V., Felice P. (2012). Posterior atrophic jaws rehabilitated with prostheses supported by 6 mm-long, 4 mm-wide implants or by longer implants in augmented bone. Preliminary results from a pilot randomised controlled trial. Eur. J. Oral Implantol..

[10] Thoma D.S., Haas R., Tutak M., Garcia A., Schincaglia G.P., Hämmerle C.H.F. (2015). Randomized controlled multicentre study comparing short dental implants (6 mm) versus longer dental implants (11–15 mm) in combination with sinus floor elevation procedures. Part 1: Demographics and patient-reported outcomes at 1 year of loading. J. Clin. Periodontol..

[11] Pjetursson B.E., Tan W.C., Zwahlen M., Lang N.P. (2008). A systematic review of the success of sinus floor elevation and survival of implants inserted in combination with sinus floor elevation. Part I: Lateral approach. J. Clin. Periodontol..

[12] Tan W.C., Lang N.P., Zwahlen M., Pjetursson B.E. (2008). A systematic review of the success of sinus floor elevation and survival of implants inserted in combination with sinus floor elevation. Part II: Transalveolar technique. J. Clin. Periodontol..

[13] Stacchi C., Lombardi T., Ottonelli R., Berton F., Perinetti G., Traini T. (2018). New bone formation after transcrestal sinus floor elevation was influenced by sinus cavity dimensions: A prospective histologic and histomorphometric study. Clin. Oral Implants Res..

[14] Younes F., Eghbali A., Goemaere T., De Bruyckere T., Cosyn J. (2018). Patient-Reported Outcomes After Lateral Wall Sinus Floor Elevation: A Systematic Review. Implant Dent..

[15] Molina A., Sanz-Sánchez I., Sanz-Martín I., Ortiz-Vigón A., Sanz M. (2022). Complications in sinus lifting procedures: Classification and management. Periodontology 2000.

[16] Starch-Jensen T., Aludden H., Hallman M., Dahlin C., Christensen A.E., Mordenfeld A. (2018). A systematic review and meta-analysis of long-term studies (five or more years) assessing maxillary sinus floor augmentation. Int. J. Oral Maxillofac. Surg..

[17] Esposito M., Hirsch J.M., Lekholm U., Thomsen P. (1998). Biological factors contributing to failures of osseointegrated oral implants. (II). Etiopathogenesis. Eur. J. Oral Sci..

[18] Kang D.-Y., Kim M., Lee S.-J., Cho I.-W., Shin H.-S., Caballé-Serrano J., Park J.-C. (2019). Early implant failure: A retrospective analysis of contributing factors. J. Periodontal Implant Sci..

[19] Chatzopoulos G.S., Wolff L.F. (2023). Dental implant failure and bone augmentation: A retrospective study. J. Clin. Exp. Dent..

[20] Jemt T., Nilsson M., Olsson M., Stenport V.F. (2017). Associations Between Early Implant Failure, Patient Age, and Patient Mortality: A 15-Year Follow-Up Study on 2566 Patients Treated with Implant-Supported Prostheses in the Edentulous Jaw. Int. J. Prosthodont..

[21] Derks J., Tomasi C. (2015). Peri-implant health and disease. A systematic review of current epidemiology. J. Clin. Periodontol..

[22] Ben-Dor A., Gabay E., Horwitz J., Zigdon-Giladi H., Machtei E.E., Mayer Y. (2021). Severe Complications Following Maxillary Sinus Augmentation Using Poly L-lactide-co-ε-caprolactone-Coated Bovine Bone: A Retrospective Study. Int. J. Oral Maxillofac. Implants.

[23] Del Fabbro M., Rosano G., Taschieri S. (2008). Implant survival rates after maxillary sinus augmentation. Eur. J. Oral Sci..

[24] Jamcoski V.H., Faot F., Marcello-Machado R.M., Melo A.C.M., Fontão F.N.G.K. (2023). 15-Year Retrospective Study on the Success Rate of Maxillary Sinus Augmentation and Implants: Influence of Bone Substitute Type, Presurgical Bone Height, and Membrane Perforation during Sinus Lift. BioMed Res. Int..

[25] Ragucci G.M., Elnayef B., del Amo F.S.-L., Wang H.-L., Hernández-Alfaro F., Gargallo-Albiol J. (2019). Influence of exposing dental implants into the sinus cavity on survival and complications rate: A systematic review. Int. J. Implant Dent..

[26] Testori T., Del Fabbro M., Bianchi F., Francetti L., Weinstein R.L., Feldman S., Vincenzi G., Sullivan D., Rossi R., Anitua E. (2002). A multicenter prospective evaluation of 2-months loaded Osseotite implants placed in the posterior jaws: 3-year follow-up results. Clin. Oral Implants Res..

[27] Wallace S.S., Froum S.J. (2003). Effect of maxillary sinus augmentation on the survival of endosseous dental implants. A systematic review. Ann. Periodontol..

[28] Summers R.B. (1994). A new concept in maxillary implant surgery: The osteotome technique. Compendium.

[29] Sakka S., Baroudi K., Nassani M.Z. (2012). Factors associated with early and late failure of dental implants. J. Investig. Clin. Dent..

[30] Masuda K., Silva E.R., Apaza Alccayhuaman K.A., Botticelli D., Xavier S.P. (2020). Histologic and Micro-CT Analyses at Implants Placed Immediately After Maxillary Sinus Elevation Using Large or Small Xenograft Granules: An Experimental Study in Rabbits. Int. J. Oral Maxillofac. Implants.

[31] Del Fabbro M., Corbella S., Weinstein T., Ceresoli V., Taschieri S. (2012). Implant survival rates after osteotome-mediated maxillary sinus augmentation: A systematic review. Clin. Implant Dent. Relat. Res..

[32] Levin L., Schwartz-Arad D. (2005). The effect of cigarette smoking on dental implants and related surgery. Implant Dent..

[33] Chrcanovic B.R., Albrektsson T., Wennerberg A. (2015). Smoking and dental implants: A systematic review and meta-analysis. J. Dent..

[34] Silverstein P. (1992). Smoking and wound healing. Am. J. Med..

[35] Sørensen L.T., Jørgensen S., Petersen L.J., Hemmingsen U., Bülow J., Loft S., Gottrup F. (2009). Acute effects of nicotine and smoking on blood flow, tissue oxygen, and aerobe metabolism of the skin and subcutis. J. Surg. Res..

[36] McMaster S.K., Paul-Clark M.J., Walters M., Fleet M., Anandarajah J., Sriskandan S., A Mitchell J. (2008). Cigarette smoke inhibits macrophage sensing of Gram-negative bacteria and lipopolysaccharide: Relative roles of nicotine and oxidant stress. Br. J. Pharmacol..

[37] Bonsmann B., Abughalia M., von See C., Dietrich T. (2025). Risk Factors for Early Implant Failure Following Sinus Augmentation: A Multi-Centre Nested Case-Control Study. J. Clin. Periodontol..

[38] Sabri H., Saleh M.H.A., Nava P., Scaini R., Testori T., Del Fabbro M. (2026). Long-term outcomes of lateral sinus floor elevation: A machine-learning analysis, systematic review, and meta-analysis of predictive factors. Periodontology 2000.

[39] Li T., Kong L., Wang Y., Hu K., Song L., Liu B., Li D., Shao J., Ding Y. (2009). Selection of optimal dental implant diameter and length in type IV bone: A three-dimensional finite element analysis. Int. J. Oral Maxillofac. Surg..

[40] Petrie C.S., Williams J.L. (2005). Comparative evaluation of implant designs: Influence of diameter, length, and taper on strains in the alveolar crest. A three-dimensional finite-element analysis. Clin. Oral Implants Res..

[41] Telleman G., Raghoebar G.M., Vissink A., den Hartog L., Huddleston Slater J.J.R., Meijer H.J.A. (2011). A systematic review of the prognosis of short (<10 mm) dental implants placed in the partially edentulous patient. J. Clin. Periodontol..

[42] Anitua E., Orive G. (2010). Short implants in maxillae and mandibles: A retrospective study with 1 to 8 years of follow-up. J. Periodontol..

[43] Schiavo-Di Flaviano V., Egido-Moreno S., González-Navarro B., Velasco-Ortega E., López-López J., Monsalve-Guil L. (2024). Influence of Schneiderian Membrane Perforation on Implant Survival Rate: Systematic Review and Meta-Analysis. J. Clin. Med..

[44] Testori T., Yu S.-H., Tavelli L., Wang H.-L. (2020). Perforation Risk Assessment in Maxillary Sinus Augmentation with Lateral Wall Technique. Int. J. Periodontics Restor. Dent..

[45] Moy P.K., Medina D., Shetty V., Aghaloo T.L. (2005). Dental implant failure rates and associated risk factors. Int. J. Oral Maxillofac. Implants.

